# miRNA in the Progression of Diabetic Kidney Disease: New Insight

**DOI:** 10.3390/ijms27010420

**Published:** 2025-12-31

**Authors:** Zhiyue Zou, Ning Zhou, Chun Zhang

**Affiliations:** Department of Nephrology, Union Hospital, Tongji Medical College, Huazhong University of Science and Technology, Wuhan 430022, China; zouzhiyue@hust.edu.cn (Z.Z.); nzhou2527@126.com (N.Z.)

**Keywords:** miRNA, diabetic kidney disease, biomarker

## Abstract

Diabetic kidney disease (DKD) is a major microvascular complication of diabetes and a leading cause of end-stage renal disease worldwide. Despite advances in metabolic and blood pressure control, the prevalence of DKD continues to rise, creating a significant clinical and socioeconomic burden. Recent studies have revealed that non-coding RNAs, particularly microRNAs (miRNAs), play an important role in the development and progression of DKD. Distinct patterns of miRNA dysregulation have been identified in specific renal cell types, including podocytes, mesangial cells, tubular epithelial cells, endothelial cells, fibroblasts, and macrophages. These alterations drive characteristic cellular injuries such as podocyte loss, mesangial matrix expansion, tubular epithelial–mesenchymal transition, endothelial dysfunction, and interstitial fibrosis. Certain miRNAs, such as miR-21, miR-192, and miR-214, reinforce profibrotic TGF-β/Smad signaling, whereas protective groups, including the miR-29 and miR-30 families, maintain epithelial stability and restrict matrix deposition. Beyond their regulatory roles, circulating and urinary miRNAs have emerged as stable, non-invasive biomarkers that reflect renal injury and disease progression. This review summarizes recent progress in elucidating cell-specific miRNA networks in DKD and highlights their potential as diagnostic indicators and therapeutic targets for precision management of diabetic kidney disease.

## 1. Introduction

Diabetic kidney disease (DKD), also known as diabetic nephropathy (DN), represents one of the most prevalent and severe microvascular complications of diabetes mellitus and remains a leading cause of end-stage kidney disease (ESKD) worldwide. Despite advances in glycemic and blood pressure control, the incidence of DKD continues to rise globally, imposing a significant socioeconomic and public health burden. Epidemiological studies estimate that approximately 20–40% of patients with diabetes eventually develop kidney impairment during the course of the disease [1]. Many of these ultimately progress to ESKD, requiring renal replacement therapy such as dialysis or kidney transplantation, which markedly reduces quality of life and increases mortality [2].

In China, the rapid increase in the diabetes prevalence has altered the etiologic landscape of chronic kidney disease (CKD). Diabetes has now surpassed glomerulonephritis as the most common cause of CKD, and its contribution to incident ESKD has markedly increased in the past two decades [3]. Recent nationwide surveys have shown that approximately one-third of Chinese adults with diabetes exhibit diabetic CKD [3], including a 30.8% prevalence of albuminuria, and a 5.5% prevalence of reduced eGFR (<60 mL/min/1.73 m^2^) [4]. These findings underscore the growing economic public health burden of DKD in Asian populations and emphasize the urgent need for earlier detection and mechanism-based interventions.

Traditionally, DKD pathogenesis has been attributed to metabolic and hemodynamic factors—including chronic hyperglycemia, hypertension, oxidative stress, and inflammation—that lead to glomerular basement membrane thickening, mesangial expansion, podocyte loss, and tubulointerstitial fibrosis. Although these processes remain central to disease pathology, they fail to fully explain the heterogeneity of the onset and progression of kidney injury among patients with comparable metabolic control. Thus, in recent years, the advent of transcriptomics and RNA-based molecular biology has brought non-coding RNAs, especially microRNAs (miRNAs), to the forefront as critical post-transcriptional regulators in the pathophysiology of DKD [5].

**Figure 1:**
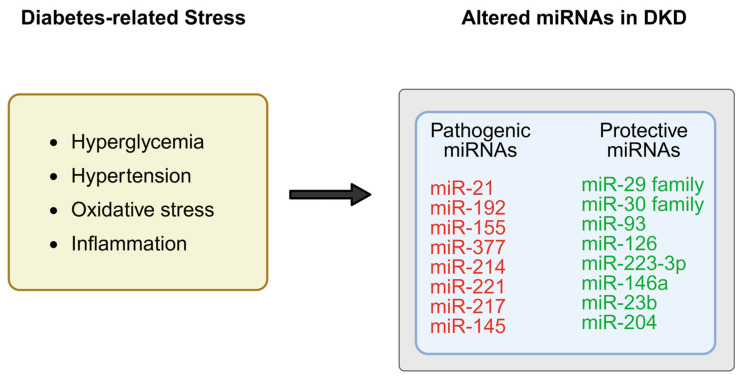
Under diabetic conditions, metabolic stress such as hyperglycemia, inflammation, and oxidative stress has been associated with dysregulation of specific microRNAs (miRNAs) in the kidney, which may promote pro-fibrotic and pro-inflammatory pathways, while protective miRNAs are downregulated, resulting in loss of inhibition on fibrotic genes and signaling cascades. Created in BioRender. Zhang, C. (2025) https://BioRender.com/8dgml63.

miRNAs are small (approximately 19–25 nucleotides in length), single-stranded non-coding RNAs that modulate gene expression through binding to complementary sequences in the 3′-untranslated regions (3′-UTRs) of target mRNAs [6], resulting in mRNA degradation or translational repression. In the kidney, miRNAs regulate a wide range of biological processes, including mesangial cell proliferation, extracellular matrix (ECM) turnover, epithelial–mesenchymal transition (EMT), tubular atrophy, inflammation, and apoptosis [7,8] (Figure 1).

Under diabetic conditions, aberrant miRNA expression contributes to the accumulation of ECM components and the activation of profibrotic signaling pathways such as transforming growth factor-β (TGF-β)/Smad [5]. For instance, miR-200a suppresses TGF-β2 expression to prevent renal fibrosis [8], while inhibition of miR-192 alleviates renal fibrosis and collagen synthesis in diabetic nephropathy [7].

Beyond their intracellular roles, circulating and urinary miRNAs have emerged as stable, non-invasive biomarkers reflecting renal injury and disease progression [5,9,10]. Abnormal expression of miR-21, miR-192, and miR-29 correlates with albuminuria, glomerular damage, and fibrosis severity. Elevated urinary miR-21 and miR-192 correlate with albuminuria and glomerular injury, while decreased miR-29 expression reflects disrupted ECM homeostasis and fibrosis progression [7,11]. Because miRNAs can be stably encapsulated within extracellular vesicles or bound to RNA-binding proteins in circulation, they resist degradation by nucleases, enabling reliable detection in biofluids [5,9]. Their remarkable stability renders them particularly suitable for early diagnosis, disease stratification, and therapeutic monitoring in DKD.

Emerging evidence suggests that the expression of miRNAs may vary across different stages of DKD, as well as between individual patients [12]. However, further studies are needed to fully elucidate these dynamic changes. For instance, miR-21 tends to be upregulated in the early phases of DKD, contributing primarily to inflammatory processes, while its sustained elevation in advanced stages is closely associated with fibrotic remodeling [13,14]. In addition, clinical studies have demonstrated that elevated serum levels of miR-217 positively correlate with proteinuria severity and DKD progression, indicating its potential utility as a stage-specific biomarker [15].

Collectively, these findings identify miRNAs as active regulators rather than passive byproducts of diabetic injury. They bridge metabolic and inflammatory stress to structural remodeling in the kidney, offering novel molecular insight and therapeutic promise [5]. Despite these advances, several challenges hinder clinical translation, including the cell-type specificity of renal miRNA expression, the difficulty in distinguishing pathogenic from compensatory changes, and the lack of standardized methods for miRNA detection and normalization. This review therefore aims to summarize recent progress in understanding the regulatory roles of miRNAs in DKD, highlighting their mechanistic functions, biomarker potential, and translational implications.

## 2. Biological Functions and Regulatory Networks of miRNAs

### 2.1. Fundamental Concepts of miRNAs

MicroRNAs (miRNAs) are small, endogenous, non-coding single-stranded RNAs of approximately 20–24 nucleotides that are evolutionarily conserved across eukaryotes [6,16,17]. They act as crucial post-transcriptional regulators by binding to complementary sequences in the 3′-UTRs of target messenger RNAs (mRNAs), leading to translational repression or mRNA degradation [18]. The specificity of miRNA–mRNA interactions is primarily determined by the “seed region” (nucleotides 2–8 of the miRNA guide strand), which recognizes partially complementary target sites and can silence translation even with imperfect base pairing [19].

A single miRNA can influence hundreds of mRNAs, and conversely, one mRNA may be targeted by multiple miRNAs, generating a complex and multilayered regulatory network [18,20,21]. This combinatorial regulation allows miRNAs to fine-tune gene expression and maintain signaling balance. By exerting coordinated yet moderate repression of multiple genes, miRNAs act as molecular rheostats rather than simple on/off switches, stabilizing transcriptional programs and cellular homeostasis [22,23,24]. These properties enable miRNAs to participate in diverse physiological processes such as differentiation, apoptosis, metabolism, and stress responses [22,25]. Dysregulation of miRNA expression disrupts these delicate networks, contributing to diverse pathological conditions, including metabolic disorders such as DKD [25,26]. Therefore, miRNAs have emerged as central regulators and potential therapeutic targets, providing new insights into disease mechanisms and treatment strategies [26].

### 2.2. Key Signaling Pathways Involving miRNAs

In DKD, miRNAs modulate a wide range of pathology pathways—fibrosis, inflammation, oxidative stress, and EMT—mainly through multiple canonical signaling pathways such as transforming growth factor-β/Smad, nuclear factor-κB (NF-κB), phosphatidylinositol-3-kinase/protein kinase B (PI3K/Akt), mitogen-activated protein kinase (MAPK), and Wnt/β-catenin [22,27].

Among these, the TGF-β/Smad pathway is the most extensively characterized fibrotic signaling axis. miR-21, one of the most studied profibrotic miRNAs, is induced by TGF-β1 and suppresses inhibitory factors including Smad7 and PTEN, thereby enhancing Smad3 activation and promoting ECM deposition [28,29,30,31]. Conversely, the miR-29 family functions as an antifibrotic regulator by directly repressing the expression of collagens and fibronectin. TGF-β1 down-regulates miR-29, releasing this inhibition and accelerating fibrogenesis [32,33]. Another critical molecule, miR-192, is also induced by TGF-β and inhibits ZEB1 and ZEB2, leading to increased collagen synthesis [34].

Inflammatory signaling mediated by NF-κB is tightly controlled by miRNAs. miR-146a serves as a negative feedback regulator by targeting IRAK1 and TRAF6, reducing NF-κB activation and limiting IL-6 and TNF-α [35]. In addition, miR-21 can indirectly activate the NF-κB signaling pathway, thereby aggravating interstitial inflammation and fibrosis in the kidney [28,29,36]. The interplay between pro-inflammatory and anti-inflammatory miRNAs fine-tunes cytokine balance and determines the renal inflammatory state.

Furthermore, miRNAs influence cell hypertrophy and ECM metabolism via the PI3K/Akt and MAPK pathways. For example, miR-214 is upregulated under high-glucose conditions, suppresses PTEN, and activates Akt signaling, which promotes mesangial-cell hypertrophy and ECM accumulation [34]. In contrast, endothelial-enriched miR-126 maintains vascular integrity and angiogenesis. Its downregulation in diabetes contributes to endothelial dysfunction and microvascular injury [37].

Aberrant activation of the Wnt/β-catenin pathway is another hallmark of DKD progression and a key driver of EMT and fibrosis. Members of the miR-200 family inhibit EMT by targeting ZEB1 and ZEB2, preserving epithelial integrity and preventing fibroblast activation, linking miRNA dysregulation to irreversible tissue remodeling [38].

The biological functions of miRNAs in DKD are highly context-dependent, varying among different renal cell types such as podocytes, mesangial cells, and tubular epithelial cells [27]. Their activity is modulated by interactions with target mRNAs, Argonaute proteins, and competing endogenous RNAs (ceRNAs) that act as molecular sponges [19,21]. Advances in transcriptomics and single-cell sequencing have revealed that miRNAs serve as nodal regulators linking metabolic stress, inflammation, and fibrosis [22,27]. Through feedback loops involving transcription factors and signaling mediators, miRNAs establish self-sustaining regulatory networks that perpetuate chronic inflammation and tissue remodeling.

These miRNA-centered networks are further integrated into key fibrotic and inflammatory signaling pathways, including TGF-β/Smad and PI3K/Akt cascades, forming complex feedback loops. For example, TGF-β signaling not only induces the expression of certain miRNAs such as miR-21 and miR-192, but is also modulated by these same miRNAs through suppression of negative regulators like Smad7 or PTEN [14,28,31]. This mutual regulatory relationship creates amplification circuits that sustain fibrotic gene expression in DKD.

Collectively, miRNAs form an integrated molecular layer that coordinates renal responses to diabetic stress, making them both powerful regulators and challenging therapeutic targets.(Table 1)

## 3. Functional Roles of miRNAs in DKD

### 3.1. miRNA Regulation and Podocyte Injury

Podocytes are highly specialized epithelial cells that form the slit diaphragm of the glomerular filtration barrier. Their structural and functional integrity is essential for maintaining selective permeability of the glomerulus. Under diabetic conditions, these cells are exposed to persistent metabolic and hemodynamic stress, leading to apoptosis, cytoskeletal disruption, and detachment from the glomerular basement membrane—hallmarks of DKD. Accumulating evidence has demonstrated that multiple microRNAs (miRNAs) play crucial roles in these pathological processes by fine-tuning gene expression involved in cytoskeletal dynamics, apoptosis, and ECM production [42,63,64].

The miR-30 family is abundantly expressed in normal podocytes and plays a crucial role in maintaining epithelial phenotype and cytoskeletal stability. Exposure to high glucose or TGF-β markedly suppresses miR-30 expression, leading to cytoskeletal instability, loss of slit-diaphragm components, and dedifferentiation of podocytes [42,64]. Mechanistic studies indicate that miR-30 targets Metadherin (Mtdh), Runx1, and Snail1, thereby repressing epithelial–mesenchymal transition and preserving structural integrity [65,66,67]. Restoring miR-30 expression can prevent glucocorticoid-sensitive podocyte injury, further confirming its essential homeostatic role [42].

Another key regulator, miR-93, links metabolic disturbance to chromatin remodeling. High glucose suppresses miR-93 expression, resulting in the derepression of mitogen- and stress-activated kinase 2 (MSK2). Elevated MSK2 phosphorylates histone H3 at serine 10, triggering transcriptional activation of profibrotic and apoptotic genes [41]. Restoration of miR-93 expression alleviates these maladaptive changes, normalizing podocyte morphology and gene expression patterns. These findings identify miR-93 as a link between metabolic stress, chromatin modification, and diabetic podocyte damage.

In contrast, miR-217 functions as a pathogenic regulator that aggravates podocyte injury through suppression of sirtuin-1 (SIRT1), an NAD^+^-dependent deacetylase that modulates oxidative stress and inflammatory signaling, and related downstream signaling pathways. Its inhibition by miR-217 leads to enhanced cell aging, inflammatory cytokine production, and fibrotic responses [15,68,69]. Increased miR-217 expression correlates with disease severity and albuminuria in patients with DKD [15]. In vitro and in vivo inhibition of miR-217, or reactivation of SIRT1, can significantly reduce podocyte apoptosis, inflammation, and ECM accumulation [68,69].

**Figure 2:**
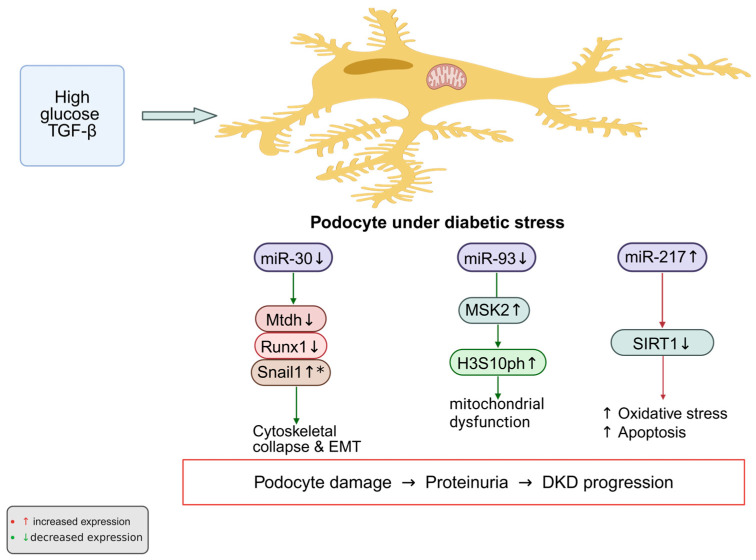
Under diabetic conditions, persistent stimulation by high glucose and TGF-β alters several miRNAs that are essential for podocyte homeostasis. The normally protective miR-30 and miR-93 are markedly reduced, whereas the pathogenic miR-217 is elevated. Loss of miR-30 leads to increased expression of its targets—Mtdh, Runx1, and Snail1—which drive epithelial–mesenchymal transition and destabilize the cytoskeleton. Downregulation of miR-93 results in accumulation of MSK2 and enhanced phosphorylation of histone H3 at serine 10 (H3S10ph), linking chromatin activation to mitochondrial injury. In contrast, elevated miR-217 suppresses SIRT1, thereby promoting oxidative stress and apoptosis. The combined effect of these miRNA alterations, as observed in experimental models, may contribute to podocyte injury and proteinuria in DKD. * Upregulation due to loss of miR-30 repression Created in BioRender. Zhang, C. (2025) https://BioRender.com/026rmif.

Taken together, these data delineate three representative miRNA axes governing podocyte fate in diabetic conditions: the miR-30 family safeguards cytoskeletal and epithelial integrity, miR-93 integrates metabolic and epigenetic stress responses, and miR-217 accelerates injury through inhibition of SIRT1 signaling. Each of these miRNAs modulates distinct yet interconnected molecular pathways controlling podocyte differentiation, cytoskeletal integrity, and stress responses. These miRNAs form a regulatory triad connecting metabolic stress to cytoskeletal and mitochondrial damage, providing mechanistic insight into podocyte failure in DKD (Figure 2).

### 3.2. miRNA Regulation and Mesangial Cell Proliferation and Fibrosis

Mesangial cells undergo abnormal proliferation and ECM deposition in response to high-glucose conditions and transforming growth factor-β (TGF-β) stimulation. These pathological alterations constitute the key histological features driving the progression of DN. Numerous studies have demonstrated that special regulate mesangial-cell phenotype by modulating proliferation, apoptosis, and ECM metabolism, often forming feedback loops with TGF-β–related pathways that amplify fibrotic responses [7,34,62,70].

Among these molecules, miR-192 has been most extensively characterized. It is highly expressed in the kidney, particularly in glomeruli, and its expression is strongly induced by TGF-β [34]. Mechanistically, miR-192 downregulates E-box repressors such as ZEB1/2 and SIP1, thereby relieving their inhibition of collagen and other ECM genes. This process reinforces TGF-β–driven expression of fibrogenic genes and promotes ECM accumulation [70]. In vivo studies using locked nucleic acid (LNA)–based antisense oligonucleotides confirmed that silencing miR-192 restores ZEB1/2 expression and decreases Col1a2, TGF-β, and fibronectin levels, thus attenuating fibrosis [7]. Similarly, antisense inhibition of miR-21 has also demonstrated renoprotective effects in diabetic mice, mitigating fibrosis and improving renal function [14,30,40,71].

miR-21, another TGF-β–responsive miRNA, promotes mesangial-cell proliferation and apoptosis resistance through repression of the PTEN/PI3K–Akt pathway [28,71]. In diabetic mouse models, its overexpression enhances ECM production and glomerular fibrosis, whereas antisense inhibition of miR-21 reduces albuminuria and ameliorates renal pathology [14,30,40,71]. These results establish miR-21 as a major driver of fibrotic progression in DN and a promising therapeutic target.

miR-377 is also upregulated under hyperglycemic conditions and contributes to ECM accumulation by increasing fibronectin expression [62]. Elevated renal miR-377 levels correlate with disease severity in both diabetic animals and patients, suggesting its involvement in glomerular sclerosis and matrix expansion [62].

Additionally, miR-214 has been identified as a fibrosis-associated miRNA that regulates autophagy, oxidative stress, and ECM metabolism. Its expression rises in parallel with miR-21, forming part of a shared “renal injury–associated miRNA signature” [43]. Mechanistic studies have shown that miR-214 inhibits autophagy through the p53/miR-214/ULK1 axis, thereby accelerating renal fibrosis in DKD [12,44]. The level of miR-214 correlates with fibrosis severity and tubular damage, indicating its diagnostic and pathogenic relevance [12,44].

Overall, TGF-β directly and indirectly regulates several miRNAs, notably miR-192 and miR-21, both of which form amplification loops reinforcing TGF-β-induced ECM gene expression [26,32,70]. Functional inhibition of these miRNAs—such as antisense blockade of miR-192 or miR-21—has been shown to mitigate fibrosis and improve renal function in experimental models [7,14,30,40]. Collectively, the miRNA–TGF-β signaling network serves as a central regulator of diabetic nephropathy pathogenesis, offering valuable insight into potential anti-fibrotic interventions.

### 3.3. miRNA Regulation and Endothelial Dysfunction

Endothelial dysfunction is a critical early event in DKD, contributing to microvascular injury and progressive glomerular damage [72,73,74]. Mitochondrial stress, oxidative imbalance, and impaired nitric oxide (NO) signaling compromise endothelial barrier integrity and lead to increased permeability of the glomerular filtration surface [72,73,74]. Multiple microRNAs have been shown to regulate these processes, influencing endothelial homeostasis and disease progression [46].

Among these regulators, miR-126 plays a pivotal role in preserving endothelial homeostasis. It supports vascular integrity by enhancing angiogenic signaling and maintaining endothelial junctions. Under diabetic conditions, miR-126 expression declines markedly, weakening endothelial repair and promoting inflammatory adhesion [46,47,48]. Clinical studies have shown that reducing circulating or urinary miR-126 levels is associated with microvascular injury and renal dysfunction in DKD patients [49]. These findings establish miR-126 as a vascular-protective miRNA with diagnostic and prognostic potential.

In contrast, miR-221 acts as a pro-apoptotic and anti-angiogenic factor. Its expression is elevated in response to high glucose, resulting in enhanced endothelial apoptosis and impaired migration through repression of the SIRT1/Nrf2 pathway [50,51]. The consequent decrease in antioxidant capacity exacerbates oxidative stress and structural disruption of the endothelium. Increased miR-221 expression correlates with endothelial injury and glomerular capillary loss, contributing to proteinuria and microvascular dysfunction in diabetic kidneys [50,51,72].

The miR-200 family—particularly miR-200b—has also emerged as a key regulator of angiogenic signaling and endothelial integrity. miR-200b modulates vascular endothelial growth factor (VEGF)–related pathways to balance angiogenesis and vascular permeability. It fine-tunes endothelial responses by targeting transcriptional repressors controlling VEGF expression [52,74]. In addition, miR-200 and miR-466 directly target Claudin-5, a key tight junction protein, thereby disrupting endothelial barrier integrity and promoting vascular leakage in diabetic conditions [53]. Evidence from diabetic retinopathy and kidney disease models supports a conserved role for miR-200b in angiogenic impairment and vascular hyperpermeability [46,52,74].

These miRNAs constitute an integrated molecular network governing the balance between endothelial protection and dysfunction in DKD. miR-126 sustains vascular protection and angiogenic function, miR-221 amplifies oxidative and apoptotic injury, while miR-200b maintains VEGF-mediated vascular balance. The dysregulation of this triad shifts endothelial cells toward dysfunction, accelerating microvascular loss and glomerular injury characteristic of diabetic kidney disease.

### 3.4. miRNA Regulation and Tubular Epithelial Cell Damage

**Figure 3:**
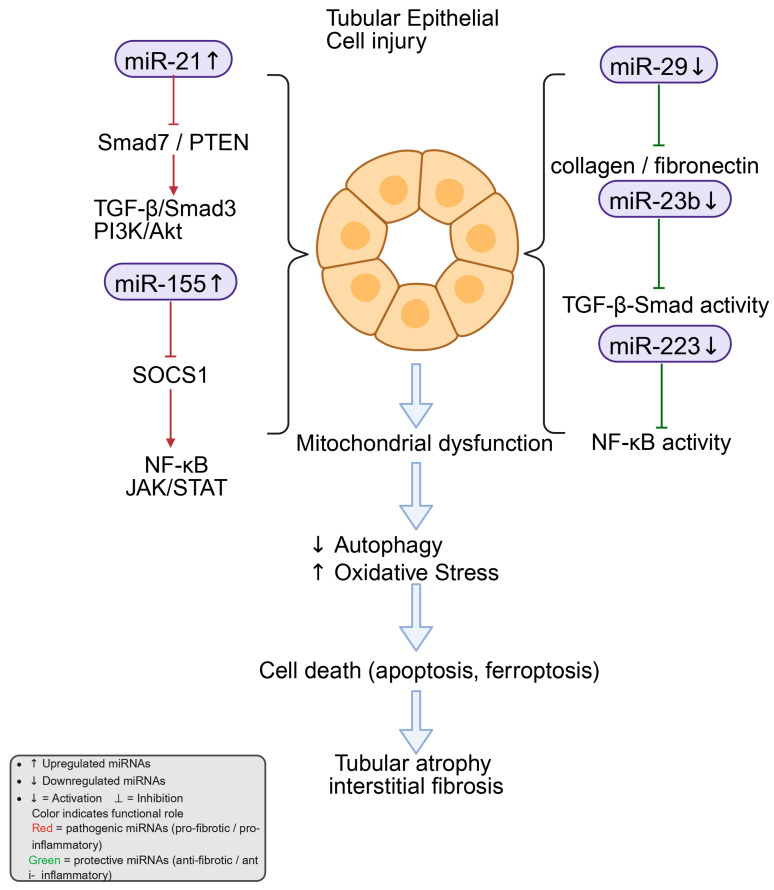
miRNA-mediated regulation of tubular epithelial cell injury in diabetic kidney disease. Exposure to high glucose and TGF-β1 changes the expression of several miRNAs in tubular epithelial cells. Among them, miR-21 and miR-155 are increased and stimulate signaling through TGF-β/Smad3, PI3K/Akt, NF-κB, and JAK–STAT, which enhance inflammation and fibrosis. In contrast, miR-29, miR-23b, and miR-223 are reduced, leading to the loss of normal control over collagen production, TGF-β activity, and NF-κB signaling. These molecular changes disturb mitochondrial function, suppress autophagy, and raise oxidative stress. The resulting injury may contribute to tubular cell death processes such as apoptosis and ferroptosis, which are implicated in tubular atrophy and interstitial fibrosis in diabetic kidney disease. Created in BioRender. Zhang, C. (2025) https://BioRender.com/vg6i6p2.

Tubular epithelial cell (TEC) injury is a central driver of DKD progression and a key antecedent of tubulointerstitial fibrosis under sustained hyperglycemia, inflammation, hypoxia, and profibrotic signaling [75]. In this context, microRNAs (miRNAs) act as post-transcriptional regulators of EMT, inflammation, extracellular matrix metabolism, and pathway crosstalk that collectively determine tubular integrity and fibrotic outcome (Figure 3).

Among these regulators, miR-21 is consistently elevated in TECs exposed to high glucose or TGF-β. It enhances TGF-β/Smad signaling by targeting negative regulators such as Smad7. Through this mechanism, miR-21 indirectly augments Smad3 activation, thereby accelerating ECM synthesis and deposition [13,14]. Experimental evidence further supports its pathophysiological relevance: silencing miR-21 in diabetic mouse models effectively ameliorates proteinuria, inflammatory infiltration, and tubulointerstitial fibrosis [40].

In contrast, the miR-29 family exerts antifibrotic control by directly repressing ECM components, including type I/IV collagens and fibronectin [11,76]. However, TGF-β1 reduces miR-29 expression, which may relieve repression of collagen genes and contribute to fibrosis; this could reflect a downstream consequence rather than a primary pathogenic mechanism [11]. Restoring miR-29b levels mitigates EMT and ECM overproduction—at least in part via inhibition of PI3K/Akt signaling—thereby preserving tubular architecture [76].

Similarly, the inflammation-related miR-155 has also been shown to play a critical pathogenic role in DKD. Its expression is significantly increased under diabetic conditions, where it amplifies NF-κB and STAT pathway activity by targeting suppressors of cytokine signaling such as SOCS1 [54,55]. This enhancement promotes pro-inflammatory cytokine production (e.g., TNF-α, IL-6) and aggravates TEC injury and apoptosis [54]. Recent work has identified a mutual regulatory loop between miR-155 and SOCS1 that sustains JAK/STAT activation, linking persistent inflammation to chronic fibrotic remodeling [55].

Moreover, miR-223-3p shows a protective profile. Reduced miR-223-3p in DKD models and patient samples is associated with epithelial damage, whereas its restoration suppresses CHUK (IKKα), limits NF-κB nuclear translocation, lowers inflammatory cytokines, and attenuates EMT marker expression [58,59].

Finally, miR-23b is downregulated in diabetic kidneys and high-glucose-treated tubular cells; re-expression curbs EMT (e.g., α-SMA upregulation) and ECM accumulation and modulates TGF-β-related networks, thereby restraining fibrotic progression [60,61].

Taken together, miR-21, miR-29, miR-155, miR-223, and miR-23b constitute a complex regulatory network orchestrating the balance between injury and repair in diabetic tubular epithelium. miR-21 and miR-155 predominantly drive profibrotic and pro-inflammatory signaling in TECs, whereas miR-29, miR-223-3p, and miR-23b function as counter-regulators that preserve epithelial features and restrain ECM deposition.

### 3.5. miRNA Regulation and Fibroblast

Activation and phenotypic transformation of renal fibroblasts are pivotal events in the development of tubulointerstitial fibrosis, which represents the final common pathway of chronic kidney injury. Activated fibroblasts express α-smooth muscle actin (α-SMA), produce large amounts of ECM proteins such as collagens and fibronectin, and increase migratory and contractile capacities [77]. Increasing evidence indicates that microRNAs serve as key post-transcriptional regulators of fibroblast activation and ECM metabolism, coordinating multiple profibrotic and antifibrotic signaling pathways in diabetic and non-diabetic kidney diseases.

Among them, miR-21 is the most extensively studied and recognized as a major pro-fibrotic miRNA. Studies in chronic kidney injury models have demonstrated that miR-21 promotes the fibroblast-to-myofibroblast transition through suppressing PTEN and Smad7, which results in sustained activation of the TGF-β/Smad3 signaling cascade, driving ECM accumulation and fibrogenic gene expression [30]. miR-21 also affects fibroblast metabolism by down-regulating mitochondrial and lipid oxidation genes, thereby shifting cells toward a glycolytic, energy-inefficient phenotype that favors fibrosis [30]. Recent reports have further revealed that tubular epithelial cells (TECs) release exosomes enriched in miR-21, which are taken up by neighboring fibroblasts and trigger activation through the PTEN/Akt pathway [78]. In a recent study, miR-21 was shown to activate the Toll-like receptor 7 (TLR7)/NF-κB pathway, promoting pro-inflammatory cytokine production and creating a positive feedback loop that reinforces fibroblast activation and chronic inflammation [79]. This dual function of miR-21—both as a direct driver of fibroblast transdifferentiation and as an amplifier of the inflammatory microenvironment—illustrates its central pathogenic role and underscores the therapeutic potential of miR-21 inhibition in mitigating renal fibrosis.

The miR-29 family functions as a critical antifibrotic regulator maintaining ECM homeostasis. TGF-β1 signaling suppresses miR-29 expression, thereby relieving its inhibitory control over ECM-related structural genes such as collagen I/IV and fibronectin, which contributes to excessive matrix deposition [11]. While this has been associated with excessive matrix deposition, it remains to be determined whether miR-29 loss is causative or a reactive adaptation to fibrotic stress. In diabetic db/db mice, restoration of miR-29b expression significantly reduced collagen accumulation and improved renal function [39]. Complementary in vitro evidence demonstrated that miR-29a-3p attenuates fibroblast activation and ECM synthesis by repressing the FOXP1-mediated TGF-β1/Smad3 pathway [80]. In addition, miR-29c has been reported to alleviate renal interstitial fibrosis by suppressing the TPM1–Wnt/β-catenin signaling axis [81]. Taken together, these results position the miR-29 family as an essential antifibrotic regulator opposing TGF-β-driven profibrotic cascades in tubular injury.

Beyond these canonical miRNAs, fibroblast regulation involves additional networks such as Rho kinase, PI3K/Akt, and other metabolic and inflammatory signals [12,82]. Furthermore, systematic analyses in CKD and progressive fibrosis models demonstrated that dysregulation of miR-21 and miR-29 appears to be a core molecular event linking these pathways and driving disease progression [83]. Interestingly, the protective function of miR-29b is not limited to fibroblasts. In podocytes, it also stabilizes mitochondrial dynamics and modulates TGF-β/Smad3 signaling, indicating shared protective mechanisms across renal cell types [33].

Overall, fibroblast activation and interstitial fibrosis are governed by a complex miRNA regulatory network in which miR-21 amplifies profibrotic and inflammatory cascades, while the miR-29 family counterbalances these effects to preserve ECM balance and renal structure.

### 3.6. miRNA Regulation and Pericyte

Pericytes are mural cells that closely surround endothelial cells in capillaries and small vessels. They are essential for maintaining microvascular stability, blood–tissue barrier integrity, and supporting endothelial survival [84,85]. In DKD, persistent hyperglycemia and chronic inflammatory cues can reduce pericyte numbers or induce phenotypic conversion toward a myofibroblast-like state. These changes weaken capillary support and accelerate interstitial fibrosis [85,86].

Accumulating evidence indicates that miRNAs constitute a key post-transcriptional regulatory layer governing pericyte activity. Among them, miR-145 is selectively enriched in pericytes and is widely regarded as a key determinant of their contractile phenotype [87]. It modulates pericyte migratory capacity and vessel-supporting functions by targeting the ETS transcription factor Fli1, which is involved in angiogenesis and vessel remodeling [86,87]. Experimental models have shown that disrupted miR-145 expression impairs pericyte coverage and microvascular stability. For example, in sepsis-induced vascular injury, altered miR-145a expression correlated with defective pericyte function and loss of capillary integrity [88]. Similar mechanisms are likely relevant to diabetic conditions, where inflammatory stress and high glucose disturb miR-145 homeostasis, weakening pericyte–endothelial communication and promoting capillary dropout [33,83,84,85].

In addition to miR-145, integrin β8 has been identified as a protective regulator of pericyte identity. It restrains pericyte-to-myofibroblast transition by suppressing the TGF-β1/TGFBR1/Smad3 signaling axis [85,86]. This interaction suggests that miRNA programs, particularly miR-145, may operate within an integrin-dependent regulatory framework to fine-tune pericyte behavior and fibrosis progression. Together, these pathways form a coordinated network in which miR-145 acts as a molecular rheostat—balancing pericyte adhesion, contractility, and migration in response to environmental stress [84,86]. When miR-145 expression remains within its physiological range, pericytes maintain normal vascular support and contribute to capillary stability. However, dysregulation of miR-145—either excessive activation or loss—can disrupt this balance, leading to vessel rarefaction and fibrotic remodeling. These findings suggest that restoring normal miR-145 activity, or enhancing complementary mechanisms such as integrin β8–mediated inhibition of TGF-β/Smad signaling, could help protect microvascular integrity and slow DKD progression [85,86,87].

### 3.7. miRNA Regulation and Monocyte–Macrophage

Monocytes and macrophages are central effectors of inflammation and immune regulation in DKD [89,90]. As highly plastic immune effectors, macrophages secrete both pro-inflammatory mediators such as TNF-α, IL-6, and MCP-1, and anti-inflammatory cytokines including IL-10, thereby regulating tissue injury and repair [90]. The relative predominance of the classically activated (M1) versus alternatively activated (M2) phenotype determines the inflammatory milieu and progression of renal fibrosis [89,90]. Increasing evidence has established that miRNAs are central post-transcriptional regulators controlling monocyte differentiation, macrophage polarization, and cytokine signaling during DKD progression.

Among these, miR-155 is a prototypical pro-inflammatory miRNA that is consistently upregulated in renal inflammation and DKD models [54,55,56,57]. It promotes inflammatory effects primarily by targeting suppressor of cytokine signaling 1 (SOCS1), thereby activating the JAK/STAT signaling cascade and enhancing NF-κB activity, thereby reinforcing M1 polarization and cytokine release [54,56,57]. Researchers demonstrated that miR-155 and SOCS1 form a mutual feedback loop, increased miR-155 reduces SOCS1 expression, and diminished SOCS1 further augments miR-155 transcription, creating an amplification circuit that aggravates renal fibrosis [54,56]. This loop represents a key molecular mechanism linking miRNA dysregulation to chronic inflammation in DKD.

In contrast, miR-146a serves as an anti-inflammatory counterbalance that limits macrophage activation [55,91]. It negatively regulates the NF-κB signaling pathway by targeting key adaptor molecules TNF receptor-associated factor 6 (TRAF6) and interleukin-1 receptor-associated kinase 1 (IRAK1), thereby dampening the downstream transcriptional activation of proinflammatory cytokines [91]. Originally identified as an NF-κB-responsive miRNA forming a negative feedback loop [91]. Subsequently, miR-146a is notably downregulated in diabetic kidney models, correlating with intensified inflammation and endothelial injury [55]. Together, the antagonistic roles of miR-155 and miR-146a exemplify the fine-tuned miRNA network that calibrates macrophage inflammatory tone and determines the balance between injury and repair in diabetic nephropathy [54,55,56].

Beyond these canonical regulators, other miRNAs such as miR-21 and miR-223 also contribute to macrophage phenotype and function. Inhibition of miR-21 attenuates macrophage-mediated inflammation and oxidative injury by upregulating heme oxygenase-1 (HO-1), thereby exerting cytoprotective effects [92]. Conversely, miR-223 has been recognized as a critical regulator of inflammation resolution and M2 macrophage polarization. Dysregulation or deficiency of miR-223 prolongs inflammatory responses and fosters fibrotic remodeling [93].

Collectively, miR-155, miR-146a, miR-21, and miR-223 constitute a coordinated molecular network that governs macrophage polarization and inflammatory responses. Through coordinated modulation of NF-κB, JAK/STAT, and antioxidant signaling cascades, these miRNAs integrate environmental cues with intracellular feedback loops to shape the trajectory of renal inflammation and fibrosis. The imbalance characterized by upregulated miR-155/miR-21 and downregulated miR-146a/miR-223 drives macrophage polarization toward a pro-inflammatory and pro-fibrotic phenotype, amplifying vascular and interstitial injury [54,55,56,92,93]. Understanding the dynamic interplay and cell-specific expression patterns of these miRNAs will be critical for developing targeted molecular interventions for DKD and other inflammatory renal disorders.

## 4. Clinical Relevance and Therapeutic Potential of miRNAs in DKD

### 4.1. miRNAs as Non-Invasive Biomarkers

MicroRNAs (miRNAs) have attracted increasing attention as promising non-invasive biomarkers due to their remarkable stability and detectability in various body fluids such as blood, plasma, and urine [94,95,96]. Unlike messenger RNA, which is prone to degradation, miRNAs maintain stability through association with Argonaute proteins or encapsulation within extracellular vesicles (EVs), protecting them from enzymatic digestion by RNases [94,95,97]. This biochemical resilience allows miRNAs to mirror organ-specific pathophysiological states. In DKD, numerous studies have demonstrated that urinary miRNA expression profiles undergo significant alterations, indicating their utility for early diagnosis, disease staging, and monitoring of therapeutic efficacy [96,98,99].

Clinical and experimental studies consistently show that urinary miR-21, miR-192, and miR-29 are markedly elevated in DKD and correlate with glomerular filtration rate decline, tubulointerstitial fibrosis, and proteinuria severity [96,98]. More recently, exosomal miRNA profiling has refined the diagnostic accuracy of urinary biomarkers. Zang et al. demonstrated that urinary exosomal miR-21-5p and miR-30b-5p can effectively distinguish early-stage DKD patients from healthy controls, implying their value as sensitive indicators for early screening [99]. Similarly, Lee et al. summarized that urinary miRNAs achieve higher diagnostic accuracy in microalbuminuria stages than conventional clinical markers [98]. These observations underscore their translational potential as molecular fingerprints of renal injury.

Urinary extracellular vesicles have been proposed as a “liquid biopsy” platform reflecting glomerular and tubular pathology in real time [100]. Consistent with this notion, Jiang et al. highlighted through meta-analysis that urinary miRNAs offer high sensitivity and specificity for the early diagnosis of DKD and could be incorporated into future clinical screening algorithms [96].

In addition to urine, circulating miRNAs in plasma also provide valuable systemic insights [94,95]. Mori et al. proposed that extracellular miRNAs act as both biomarkers and signaling molecules that participate in inter-organ communication. Moreover, studies have confirmed the remarkable stability of circulating miRNAs in stored plasma samples. Sanz-Rubio et al. reported that exosomal miRNAs preserve their integrity across multiple storage conditions and repeated freeze–thaw cycles, confirming their robustness for clinical application [101]. Building on this, Glinge et al. emphasized that pre-analytical factors—such as blood collection, RNA extraction, and storage procedures—can markedly influence quantitative outcomes and overall reproducibility [102]. These findings collectively indicate that, beyond biological stability, the reproducibility of circulating miRNA measurements relies heavily on standardized laboratory workflows. Therefore, the establishment of standardized workflows is crucial to ensure the analytical reliability of circulating miRNA-based biomarkers.

Downregulated miRNAs also hold diagnostic relevance. Guo et al. found that plasma miR-204 levels are markedly associated with glomerulosclerosis and inflammatory markers, suggesting that miR-204 may serve as an early predictor of renal functional decline [103]. Motshwari et al. further summarized that miRNA signatures transcend specific etiologies, offering a unified molecular framework for distinguishing among diverse renal diseases and stages of progression [104]. These studies collectively support the concept that both elevated and diminished miRNAs can provide complementary diagnostic information, reflecting the multifaceted nature of renal pathophysiology [103,104].

Collectively, these studies highlight that both increased and decreased miRNAs can provide complementary diagnostic value. Despite such promise, challenges remain—particularly in normalization methods, pre-analytical variability, and multicenter reproducibility [96,102]. Establishing standardized workflows is therefore essential for clinical translation.

### 4.2. Advances in miRNA Mimics and AntagomiRs

Therapeutic modulation of miRNAs through mimics and inhibitors represents an emerging frontier in RNA-based medicine [12,105,106,107]. miRNA mimics replenish lost or downregulated molecules, whereas antagomiRs, chemically stabilized antisense oligonucleotides, suppress overexpressed pathogenic miRNAs [108,109,110]. This complementary strategy allows restoration of balanced post-transcriptional control disrupted during DKD progression [107].

Preclinical renal-disease models have highlighted the therapeutic potential of both approaches. In DKD and other fibrotic nephropathies, inhibition of miR-21—a well-characterized pro-fibrotic miRNA—has been shown to ameliorate glomerulosclerosis, reduce extracellular-matrix deposition, and attenuate renal fibrosis [12,111,112]. Mahtal et al. demonstrated that miR-21 inhibition in murine DKD models significantly decreased TGF-β signaling activity and improved renal histopathology [12]. Conversely, restoring protective miR-29 levels via synthetic mimics has been shown to normalize ECM turnover and reduce collagen and fibronectin accumulation [112,113]. O’Reilly emphasized that while such mimicry can effectively reverse fibrosis, challenges remain in achieving efficient and durable delivery [112].

In parallel, the exploration of extracellular vesicles—particularly exosomes—as delivery vehicles for miRNA therapeutics has gained momentum. Exosome-mediated delivery systems offer unique advantages due to their biocompatibility, ability to evade immune detection, and intrinsic capacity for tissue-specific targeting. Studies in renal models have demonstrated that exosome-loaded miRNA mimics can selectively target renal tubular or mesangial cells, achieving local therapeutic effects while minimizing systemic exposure and toxicity [111,114,115]. The clinical feasibility of miRNA mimic therapy was first validated through MRX34, a liposomal formulation of a synthetic miR-34a mimic. In the first-in-human trial, MRX34 successfully achieved dose-dependent suppression of its target genes in patient blood cells and tumor biopsies, demonstrating on-target efficacy in vivo [116,117]. However, the associated immune-related adverse events, including cytokine release and hepatotoxicity, highlighted the importance of optimized formulations and immune modulation [116].

On the inhibitory side, Miravirsen, an LNA-modified anti-miR-122, demonstrated durable target suppression and viral load reduction in hepatitis C patients, providing proof-of-concept for systemic anti-miR therapy [118]. More recently, a first-in-class LNA-based anti-miR-221 compound entered early-phase human testing, confirming the clinical feasibility of LNA backbones and further validating their therapeutic potential [119].

Recent advances in molecular design and delivery systems have substantially expanded the therapeutic scope of miRNA-based drugs. Lipid nanoparticles (LNPs), circular or tri-armed RNA scaffolds, and aptamer-mediated targeted delivery systems have all been engineered to enhance the stability, tissue enrichment, and cellular uptake of miRNA mimics and inhibitors [108,110,114]. For instance, Alsenousy et al. developed a three-way junction (3WJ) aptamer-guided anti-miR construct that preferentially accumulates in renal tissue, markedly enhancing antifibrotic efficacy in experimental chronic kidney disease while limiting systemic immune activation [114]. Similarly, innovations in nanoparticle formulation and RNA chemistry—including phosphorothioate linkages and 2′-O-methyl or locked-nucleic-acid modifications—have proven effective in reducing nuclease degradation and off-target immunostimulation [106,108,120].

Despite these promising developments, miRNA therapeutics remain in the early translation stages. Key translational barriers include determining the optimal dosing range, minimizing innate immune activation, achieving sustained bioavailability, and ensuring tissue-specific delivery without systemic toxicity. Long-term safety profiles remain under evaluation, emphasizing the need for careful risk assessment. Nevertheless, continuous improvements in chemical modification, formulation stability, and delivery precision are progressively addressing these limitations. With the maturation of nanotechnology and RNA engineering, these therapeutic platforms are expected to evolve into clinically viable strategies for targeted, durable, and safe treatment of renal diseases [12,106,108,114].

### 4.3. miRNA-Targeted Interventions for DKD

The therapeutic success of miRNA-based interventions in DKD depends on the rational design of oligonucleotide chemistry and the development of efficient delivery systems that enable selective accumulation within diseased renal tissues [12,105,106]. Both the physicochemical properties of the therapeutic molecule—whether a mimic or an inhibitor—and the delivery vehicle determine its stability, biodistribution, and pharmacological precision. Recent developments in nanomedicine have generated diverse delivery technologies, including LNPs, branched or circular nucleic acid scaffolds, exosome-based vesicles, and kidney-targeted ligands, all aiming to improve in vivo stability and minimize systemic toxicity [106,108,114,120].

Among these strategies, LNPs and cyclic or tri-armed nucleic acid architectures have demonstrated improved serum stability and cellular uptake efficiency, thereby supporting intravenous systemic administration of miRNA therapeutics [105,108,120]. Targeted delivery remains an essential requirement for clinical translation. Aptamer-conjugated or receptor-directed systems confer molecular “addressing” capability and markedly increase renal cell specificity [108,114].

In parallel, exosome carriers are gaining interest as natural delivery vehicles because of their natural compatibility, low immunogenicity, and intrinsic ability to traverse biological barriers. Notably, exosomes derived from renal or urinary sources exhibit regulated miRNA content reflective of their cell of origin, offering experimental evidence for “organ-derived EVs” as potential vehicles for targeted miRNA re-delivery to the kidney [110,121].

Despite encouraging preclinical efficacy, several translational barriers persist [12,105,106]. Major challenges include inefficient in vivo delivery, rapid degradation in circulation, and limited tissue specificity [122]. Systemically administered miRNA mimics or inhibitors may provoke off-target effects and innate immune activation through Toll-like receptor or complement pathways, thereby limiting the tolerated dose range [123]. Additionally, miRNAs are susceptible to rapid clearance and enzymatic degradation, while unmodified delivery systems often result in poor stability and off-target activity [123,124]. To improve molecular stability and reduce immunogenicity, chemical modifications such as 2′-O-methylation, phosphorothioate linkages, and locked nucleic acid (LNA) incorporation have been widely applied [108,119,120,123]. In addition, renal-targeting strategies based on megalin/cubilin endocytosis or podocyte-specific ligands help enhance tissue specificity and reduce systemic exposure [108,114]. While novel carriers such as exosomes, chitosan nanoparticles, and immunoliposomes have shown promise in improving renal targeting and stability [125], challenges related to immune response, biodistribution, and endosomal escape still need to be addressed [122]. Addressing these limitations is critical for advancing miRNA-based therapies toward clinical application in DKD.

Although preclinical data have demonstrated substantial reductions in glomerulosclerosis, inflammation, and fibrosis after miRNA modulation, clinical translation is still in an early phase. Recent evidence underscores that miRNAs such as miR-29b, when delivered using biocompatible carriers like chitosan nanoparticles, exert broad therapeutic effects on both renal fibrosis and systemic complications, including hypertension, highlighting their multi-target potential [126]. Furthermore, immune-modulating miRNAs, such as miR-126, have shown endothelial-protective effects when encapsulated in immunoliposomes, indicating a feasible path toward tissue-targeted delivery with anti-inflammatory outcomes [125]. The main challenges include achieving sustained renal bioavailability, maintaining long-term safety, and optimizing delivery efficiency. Future research should emphasize kidney-specific delivery strategies directed to proximal tubular and podocyte populations, as well as combination therapies integrating anti-fibrotic or immunomodulatory agents to enhance efficacy. Equally important is the development of companion diagnostic frameworks utilizing circulating or urinary miRNAs as biomarkers to monitor therapeutic response and guide patient selection. Integrating these diagnostic and therapeutic paradigms will be key to transforming miRNA therapy into a viable precision-medicine modality for DKD and other chronic kidney diseases.

## 5. Conclusions and Perspectives

MicroRNAs have emerged as central regulatory nodes orchestrating the molecular networks that underlie the onset and progression of DKD. Through post-transcriptional control across key renal cell populations—podocytes, mesangial cells, tubular epithelial cells, endothelial cells, fibroblasts, and macrophages—miRNAs coordinate the balance between injury and repair within the diabetic kidney. Their dysregulation has been linked to glomerulosclerosis, tubular atrophy, and interstitial fibrosis in preclinical models, while facilitating intercellular communication via exosomes and other extracellular vesicles. These findings support the role of miRNAs as active regulators in renal pathology. However, whether such dysregulation reflects a causal mechanism or a compensatory response to injury remains to be clarified, particularly in human studies.

Clinically, miRNAs exhibit remarkable stability and detectability in body fluids, which makes them promising non-invasive biomarkers for DKD. Altered urinary and circulating miRNAs, particularly miR-21, miR-192, and miR-29, correlate strongly with albuminuria, glomerular filtration decline, and histologic fibrosis. Their reproducible expression patterns across multiple studies underscore their potential roles in early diagnosis, disease stratification, and therapeutic monitoring. At the same time, miRNA-based therapeutics—such as mimics restoring downregulated species and antagomiRs inhibiting profibrotic miRNAs—have demonstrated therapeutic potential in preclinical DKD models, reducing proteinuria, inflammation, and fibrosis. Early clinical experiences, including MRX34 (a miR-34a mimic) and Miravirsen (an anti-miR-122 compound), have provided preliminary evidence supporting the feasibility of miRNA modulation in humans, though immune activation and delivery specificity remain major translational barriers.

Future efforts should focus on several key directions. First, deeper elucidation of the integrated miRNA–mRNA–lncRNA–circRNA regulatory network is required to clarify multilayered mechanisms of signal transduction and cell–cell communication in DKD. Second, the establishment of standardized, reproducible analytical platforms for miRNA quantification—across different laboratories and populations—is critical for reliable biomarker validation. Third, continued refinement of kidney-specific delivery systems, including exosome-based and nanoparticle-based vehicles, will enhance therapeutic precision and minimize systemic exposure. Finally, combinatorial strategies coupling miRNA-targeted therapy with anti-fibrotic or immunomodulatory agents may further improve efficacy and broaden clinical applicability.

Looking ahead, the clinical translation of miRNA therapeutics in DKD will depend on their integration into broader therapeutic frameworks. Combination strategies—such as the co-administration of miRNA-based drugs with SGLT2 inhibitors, which offer renal benefits beyond glucose regulation—may enhance overall efficacy [127]. Moreover, the application of nanomedicines, including exosomes and liposomal carriers, enables kidney-specific delivery while minimizing off-target effects, improving therapeutic precision [122]. Trials exploring these modalities are ongoing, yet their incorporation into clinical protocols requires standardized biomarker systems and clear physician awareness. Recent guidance highlights that clinicians must understand not only the diagnostic utility of miRNAs but also their evolving therapeutic roles in chronic kidney disease [124]. Thus, future development should prioritize long-term safety validation, delivery optimization, and combination therapy design to establish miRNAs as effective and sustainable treatment options in DKD.

## Figures and Tables

**Table 1:** Cell-Type-Specific Roles of miRNAs in DKD Pathogenesis.

miR	Main Targets/Pathways	Cell Response	Outcome	Evidence Level	Reference
miR-192	⊣ ZEB1/2 → TGF-β/Smad signaling	→ Mesangial ECM production	→ Collagen accumulation and fibrosis	Cellular, Animal, Human observational	[7,34]
miR-29 family	TGF-β1 ⊣ miR-29 → Col I/IV, FN1 derepression	→ Tubular EMT and ECM synthesis	→ Interstitial fibrosis	Cellular, Animal, Human observational	[11,32,39]
miR-21	⊣ Smad7/PTEN → TGF-β/Smad3 and PI3K-Akt	→ Fibroblast activation and metabolic reprogramming	→ Fibrosis and proteinuria	Cellular, Animal, Human observational	[14,30,40]
miR-200a	⊣ TGF-β2	⊣ EMT	⊣ Renal fibrosis	Cellular only	[8]
miR-93	⊣ MSK2 ⊣ H3S10ph	⊣ Podocyte stress → Morphology restoration	⊣ Proteinuria and injury	Cellular, Animal	[41]
miR-30 family	⊣ Mtdh/Runx1/Snail1; HG/TGF-β ⊣ miR-30	⊣ Cytoskeleton stability	→ Podocyte loss and proteinuria	Cellular, limited human expression data	[42]
miR-214	⊣ PTEN → Akt activation; ⊣ ULK1	→ Mesangial hypertrophy; ⊣ Autophagy	→ Fibrosis progression	Cellular, Animal, Human observational	[43,44,45]
miR-126	Diabetes ⊣ miR-126	⊣ Endothelial repair → Vascular instability	→ Microvascular injury	Human observational, limited in vitro	[37,46,47,48,49]
miR-221	⊣ SIRT1/Nrf2	→ Endothelial apoptosis; ⊣ Migration	→ Dysfunction and oxidative stress	Cellular, Animal, Human observational	[50,51]
miR-200b	→ VEGF pathway modulation	→ Endothelial permeability imbalance	→ Leakage and vascular injury	Cellular, Animal, indirect human evidence	[52]
miR-466 family	⊣ Claudin-5	→ Tight junction disruption	→ Vascular leakage and damage	Cellular only	[53]
miR-155	⊣ SOCS1 → JAK/STAT and NF-κB activation	→ Epithelial/endothelial inflammation	→ Fibrosis progression	Cellular, Animal, Human observational	[54,55,56]
miR-146a	⊣ IRAK1/TRAF6 ⊣ NF-κB	⊣ Inflammatory signaling	⊣ Renal inflammation	Cellular, Animal, Human observational	[55,57]
miR-223-3p	⊣ CHUK (IKKα) ⊣ NF-κB translocation	⊣ Tubular inflammation and EMT	⊣ Interstitial fibrosis	Cellular, Animal, Human observational	[58,59]
miR-23b	⊣ TGF-β-induced EMT signaling	⊣ EMT and ECM expression	⊣ Fibrosis	Cellular, Animal	[60,61]
miR-377	→ FN1 expression	→ Mesangial ECM accumulation	→ Glomerulosclerosis	Cellular, Animal, Human observational	[62]

→ indicates activation or upregulation; ⊣ indicates inhibition or suppression of targets or pathways. Abbreviations: ECM, extracellular matrix; EMT, epithelial–mesenchymal transition. Evidence levels were classified as: Cellular: in vitro mechanistic studies in renal cells; Animal: in vivo rodent models; Human observational: cross-sectional or correlational findings in patients. Additionally, as most human studies are observational, miRNA changes may represent adaptive or compensatory responses rather than primary disease drivers.

## Data Availability

No new data were created or analyzed in this study.
